# In vivo miR-138-5p inhibition alleviates monocrotaline-induced pulmonary hypertension and normalizes pulmonary KCNK3 and SLC45A3 expression

**DOI:** 10.1186/s12931-020-01444-7

**Published:** 2020-07-16

**Authors:** Hélène Le Ribeuz, Audrey Courboulin, Maria-Rosa Ghigna, Mélanie Lambert, Aurélie Hautefort, Marc Humbert, David Montani, Sylvia Cohen-Kaminsky, Frédéric Perros, Fabrice Antigny

**Affiliations:** 1 Faculté de Médecine, Université Paris-Saclay, Le Kremlin-Bicêtre, France; 2 INSERM UMR_S 999 « Hypertension pulmonaire : Physiopathologie et Innovation Thérapeutique », Hôpital Marie Lannelongue, Le Plessis-Robinson, France; 3 Assistance Publique - Hôpitaux de Paris (AP-HP), Service de Pneumologie et Soins Intensifs Respiratoires, Centre de Référence de l’Hypertension Pulmonaire, Hôpital Bicêtre, Le Kremlin-Bicêtre, France

**Keywords:** miR-138, PAH, KCNK3, Proliferation, SLC45A3

## Abstract

**Background:**

The pathogenesis of pulmonary arterial hypertension (PAH) involves many signalling pathways. MicroRNAs are potential candidates involved in simultaneously coordinating multiple genes under such multifactorial conditions.

**Methods and results:**

MiR-138-5p is overexpressed in pulmonary arterial smooth muscle cells (PASMCs) from PAH patients and in lungs from rats with monocrotaline-induced pulmonary hypertension (MCT-PH). MiR-138-5p is predicted to regulate the expression of the potassium channel KCNK3, whose loss is associated with the development and progression of PAH. We hypothesized that, in vivo, miR-138-5p inhibition would restore KCNK3 lung expression and subsequently alleviate PAH.

Nebulization-based delivery of anti-miR-138-5p to rats with established MCT-PH significantly reduced the right ventricular systolic pressure and significantly improved the pulmonary arterial acceleration time (PAAT). These haemodynamic improvements were related to decrease pulmonary vascular remodelling, lung inflammation and pulmonary vascular cell proliferation in situ. In vivo inhibition of miR-138-5p restored KCNK3 mRNA expression and SLC45A3 protein expression in the lungs.

**Conclusions:**

We confirmed that in vivo inhibition of miR-138-5p reduces the development of PH in experimental MCT-PH. The possible curative mechanisms involve at least the normalization of lung KCNK3 as well as SLC45A3 expression.

## Background

Pulmonary arterial hypertension (PAH) is a lethal cardiopulmonary disease with a multifactorial and complex pathogenesis that is characterized by an elevation in mean pulmonary arterial pressure (mPAP) greater than or equal to 20 mmHg and by an increase in pulmonary vascular resistance (PVR) greater than 3 Woods units ^1^. Increased mPAP is due to increased PVR, which is a consequence of the occlusive remodelling of distal pulmonary arteries. This occlusion ultimately leads to right ventricular (RV) hypertrophy and RV failure [1]. Pulmonary vascular remodelling is mainly based on the aberrant proliferation and apoptosis resistance of vascular cells, including pulmonary artery endothelial cells (PAECs) and pulmonary arterial smooth muscle cells (PASMCs) [2]. MicroRNAs (miRNAs) regulate processes involved in the development and progression of PAH [3]. MiRNAs are small noncoding RNA molecules that contain approximately 19–21 nucleotides. The interaction of miRNAs with 3′ UTRs leads to the cleavage of target mRNAs or to translational repression, resulting in decreased expression of the related proteins. Dysregulation of miRNA expression is involved in many diseases, ranging from several cardiovascular diseases to cancers [4]. The pathogenesis of PAH has similarities with that of cancers, such as excessive cell proliferation, apoptosis resistance, metabolic shifts, or phenotypic transition [5]. In PAH, the dysregulation of several miRNAs is involved in the aberrant proliferation of PASMCs and dysfunction of PAECs [3, 4]. In 2011, Courboulin et al. analysed the expression of 377 different miRNAs in PASMCs from idiopathic PAH (iPAH) patients. The authors identified seven miRNAs that are aberrantly expressed in PASMCs from iPAH patients (miR-204, miR-450a, miR-145 miR-302b, miR-27b, miR-367, and miR-138-5p) [6]. As in PASMCs from human iPAH patients, miR-138 expression is increased in PASMCs from human non-PAH individuals after exposure to hypoxia. Moreover, exogenous miR-138-5p (miR-138 mimic) inhibits apoptosis in PASMCs [7]. Liu J-J et al. recently reported that miR-138-5p promotes proliferation and suppresses mitochondrial depolarization in human PASMCs by reducing KCNK3 expression [8]. Mutations in the *KCNK3* gene are responsible for the first channelopathy identified in PAH [9–12]. Moreover, we found that KCNK3 dysfunction in the pulmonary vasculature and RV levels are hallmarks of PAH [13, 14]. The *KCNK3* gene encodes an outward-K^+^ channel that is a member of the two-pore K^+^ channel (K2P) family, and this channel is also known as TASK-1 (Twik-related-acid-sensitive-K^+^ channel) or K2P3.1 [15, 16]. Finally, the in vivo inhibition of miR-138-5p was found to reduce the development of severe experimental PAH by restoring the expression of the lung mitochondrial calcium uniporter (MCU) complex (MCUC) [17]. In this study, we hypothesized that the in vivo inhibition of miR-138-5p reduces the development of experimental PH, partly by restoring the expression and function of KCNK3 in the lungs.

## Material and methods

### Chemicals

Monocrotaline (MCT) was obtained from Sigma. Anti-miR-138-5p and anti-miR-Control were obtained from Dharmacon.

### Animals

The animal facility is licensed by the French Ministry of Agriculture (agreement N° C92–019-01). This study was approved by the Committee on the Ethics of Animal Experiments (CEEA26 CAP Sud). The animal experiments were performed conforming to the guidelines from Directive 2010/63/EU on 22 September 2010 of the European Parliament on the protection of animals used for scientific purposes and complied with the French institution’s guidelines for animal care and handling.

### Pulmonary hypertension induction

Male Wistar rats (150 g) were used and maintained in a temperature-controlled room with a 12:12 light–dark cycle. The in vivo experimental design was as follows: pulmonary hypertension was induced in the rats by a single injection of monocrotaline (MCT; 60 mg/kg, s.c.). MCT was dissolved in HCl (1 N) neutralized with NaOH (1 N).

### In vivo miRNA delivery to the lung

MCT-exposed rats were treated either with anti-miR-138-5p or with anti-miR-Control (100 μmol/L of saline solution, twice a week by intratracheal nebulization) at D14 and D18 in a curative protocol. Rats were anesthetized with a ketamine-xylazine mix (respectively, 75–10 mg/kg and 0.3 mL/100 g, i.p.) and intubated orotracheally. Anti-miR-138-5p and anti-miR-Control were nebulized using an Aeroneb® Lab Control and Aeroneb® Lab Nebulizer Unit (Kent Scientific Corporation). Rats were allowed to recover from anesthesia.

### Haemodynamic measurements

Hemodynamic measurements were performed 21 days after MCT-exposure. The rats were placed under general anaesthesia and spontaneous breathing with an Isoflurane Rodent Anesthesia System (Minerve, Esternay, France) (maintenance: isoflurane 2% in room air). The haemodynamic measurements, such as right ventricular systolic pressure (RVSP; mmHg) and cardiac output (CO; mL/min), were performed prior to tissue collection, as previously described [13]. After evaluation of RVSP and CO, we introduced a catheter into carotid artery to measure systemic blood pressure. All analyses were performed blinded: rats’ experimental conditions were unknown by the operator during catheterization and data interpretation. PVR were evaluated by calculating the RVSP / CO ratio. For Fulton’s index of right ventricular (RV) hypertrophy, the ratio of RV weight to left ventricular (LV) plus septal (S) weight (RV/LV + S) was calculated.

### Echocardiographic measurement

The evaluation by transthoracic echocardiography (TTE) was performed with a digital ultrasound system (Vivid E9, GE Healthcare) by using a high-frequency phased array transducer (12 S-D 4–12 MHz, GE Healthcare) at D14 and D21 after MCT-exposure. The echocardiographic evaluation procedure was performed under general anaesthesia and spontaneous breathing with an Isoflurane Rodent Anesthesia System (Minerve, Esternay, France) (maintenance: isoflurane 2% in room air). The rats were shaved, and the body temperature was controlled during the experiments. All the analyses were performed in a blinded fashion, that is, the rats’ experimental conditions were unknown by the operator during the TTE examination and data interpretation. The measurements of pulmonary artery acceleration time (PAAT), heart rate (HR) and pulmonary artery velocity time integral (VTI) were performed as previously described [18].

### Reverse transcription quantitative PCR (RT-qPCR)

Total RNA was extracted using TRIzol reagent according to the standard procedures from frozen lung collected from Control, MCT-miR-Control and MCT-miR-138-5p treated animals. To remove the genomic DNA contamination from the RNA preparations before reverse transcription, mRNA was treated with DNAse (Qiagen, Valencia, CA, USA; cat. no. 205311). Then, one microgram of total RNA was reverse-transcribed using the QuantiTect Reverse Transcription Kit (Qiagen, Valencia, CA, USA; cat. no. 205311). The gene expression was quantified using qPCR following the standard protocol for ready-to-use TaqMan gene expression assays (Life Technologies) on a StepOne Plus Real-Time PCR System (Life Technologies). The predesigned probe sets used for experiments were as follows: Rat *Kcnk3*: Rn04223042_m1, Rat *β-Actin*: Rn00667869_m1. Fold change of RNA expression was calculated using the formula (2 − ^ΔΔCt^), normalized to *β-Actin* expression.

### Quantitative RT-PCR of mature miRNAs

Mature miRNA expression was evaluated using TaqMan MicroRNA Assays (Thermo Fisher Scientific) and the Applied Biosystems StepOne+ Real Time PCR device (Thermo Fisher Scientific). Expression levels were normalized to U6 and calculated using the comparative Ct method (2 − ^ΔΔCt^). In order to ensure biological relevance, only Ct values < 35 were utilized in the analysis. U6snRNA: 001973 and miR-138-5p: 002284.

### Western blot analysis

The western blot protocol was conducted as previously described [18]. The antibodies used in the study were as follows: anti-CD45 (BD Biosciences, 610266, (1/1000), anti-ROCK2 (Cell Signaling, 1/1000), SLC45A3 (Abcam, ab137065, 1/1000), and anti-ALDH1A2 (Abcam, ab156019, 1/2000). Unfortunately, there is no available specific antibodies for KCNK3 [19, 20], thus avoiding any measurement of the protein expression. When tested in kcnk3-knockout miceand Kcnk3-mutated rats, we previously found that all commercially anti-KCNK3 antibodies tested were unusable [20]. This is why we analyzed KCNK3 expression by RT-qPCR. The blots were incubated with horseradish peroxidase (HRP)-conjugated goat anti-mouse diluted 1:10,000 (Cell Signaling) or with HRP-conjugated goat anti-rabbit diluted 1:5000 (Cell Signaling). The antibodies were detected using ECL reagents. (Perkin–Elmer). ImageJ software was used to quantify the level of protein expression.

### In situ cell proliferation

Cell proliferation in the rats was analysed by quantifying the pulmonary cells that incorporated the exogenously supplied 5-ethynyl-2′-deoxyuridine (EdU) to identify the cells undergoing DNA replication. EdU was intraperitoneally injected into the rats at 50 μg/g 24 h before sacrifice [21]. EdU staining was performed using the Click-iT EdU imaging kit (C10337, Life Technologies) on frozen sections according to the manufacturer’s instructions. To characterize the EdU-positive cells, we performed double-immunostaining with FITC-labelled anti-α-SMA (clone 1A4, Sigma-Aldrich, Lyon, France, 1/100) or with anti-CD34 (EP373Y, Abcam, Amsterdam, 1/200). Images were captured with an LSM 700 confocal microscope (Carl Zeiss, Le Pecq, France). Images were recorded and analysed with ZEN software (Carl Zeiss).

### Pulmonary vessel remodelling analysis

The lungs were fixed in 4% paraformaldehyde, embedded in paraffin and serially sectioned (5 μm). The histopathological evaluation of the lungs was performed by a pulmonary pathologist who was blinded to the genotypes and treatment group assignments of the rats. The morphometric analyses were performed on sections stained with haematein-eosin-safran (HES). The lung samples from all the conditions were analysed by conventional light microscopy using quantitative semiautomated software (NIS-BR; Nikon, Champigny sur Marne, France). Vascular remodelling was assessed in all the pulmonary vessels larger than 50 μm and less than 100 μm that were identified in 20 randomly selected microscopic fields per tissue section. The wall thickness was calculated according to the following equation: (External diameter -Internal diameter) / (External diameter)) × 100. Images were captured at 40X magnification with a Nikon Eclipse 80i microscope.

Orcein staining was also performed to visualize the elastic fibres within the pulmonary vessels. The pulmonary arteries were identified by the presence of two elastic lamina, and then, we measured the medial wall thickness (in μm) as MWT = external elastic lamina [EEL] - internal elastic lamina [IEL]. Images were captured at 40X magnification with a Nikon Eclispe 80i microscope.

### Immunohistochemistry

The paraffin-embedded, 5-μm-thick sections of the rat lungs were mounted on SuperfrostPlus slides (Thermo Scientific, Villebon sur Yvette, France). Immunostaining using anti-CD3 (SP7 rabbit monoclonal) and anti-CD68 (KP1 mouse monoclonal) antibodies was performed according to the automated protocol of the facility (benchmark GX autostainer, Ventana Medical System, Roche). The number of CD68-positive or CD3-positive cells in the lung samples from all the conditions was analysed by conventional light microscopy using quantitative semiautomated software (NIS-BR; Nikon, Champigny sur Marne, France). Images were captured at 40X magnification with a Nikon Eclipse 80i microscope.

### Immunofluorescence staining

The frozen lung sections (6 μm) were air dried. The sections were fixed with paraformaldehyde (PFA 4%) for 10 min at room temperature. The free aldehyde groups from PFA fixation were quenched for 10 min with 50 mM NH4Cl solution. The sections were blocked with human (10%) or rat (10%) serum in PBS for 1 h at room temperature. We used primary antibodies against VWF (1/400, Dako A0082) and α-smooth muscle actin (α-SMA, Sigma, F3777) (1/200). Detection of the primary antibodies was performed with goat anti-rabbit (1/200) secondary antibodies. The slides were counterstained with 4′,6′-diamidino-2-phenylindole (DAPI).

### miR-138-5p-regulated gene identification

To identify the main miR-138-5p-regulated gene, we used Ingenuity Pathway Analysis (Qiagen, Redwood City, CA) as previously described [22].

### Statistical analyses

All the statistical tests were performed using GraphPad Prism software (GraphPad, version 6.0 for Windows). All the data were verified for normal distribution. All the values are reported as the mean ± S.E.M. For all the experiments, the difference between two groups was assessed by two-tailed unpaired Student’s test. The difference among at least three groups was assessed with one-way analysis of variance completed by Dunnett’s least significant difference post hoc test for multiple comparisons. The differences were considered statistically significant at *p*-values < 0.05.

## Results

### MiR-138-5p expression is increased in the lungs of MCT-exposed rats

**Fig. 1:**
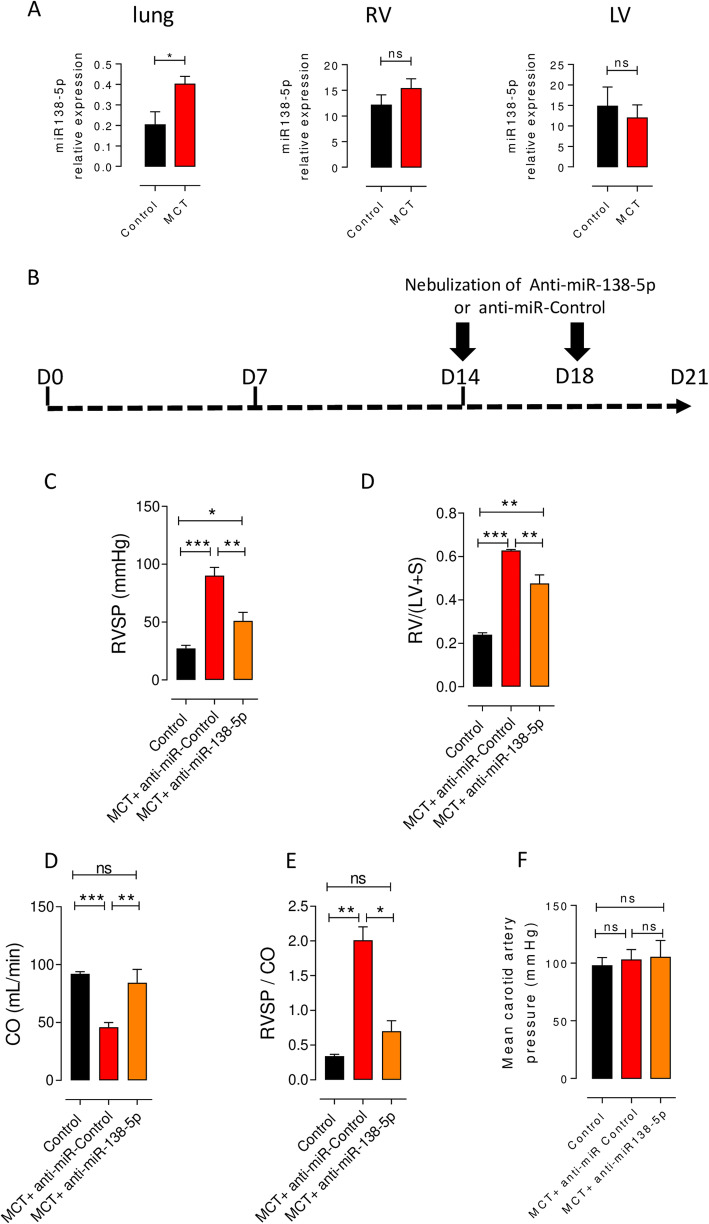
In vivo *inhibition of miR-138-5p reverses PH.***a** miR-138-5p expression in the lung (left panel), right ventricle (RV, middle panel) and left ventricle (LV, right panel) of control rats and rats with MCT-PH (3 weeks after MCT exposure). **b** Protocol for the administration of anti-miRs in rats with established PH. Fourteen days after the administration of monocrotaline (MCT, 60 mg/kg), the rats were anaesthetized and nebulized 2 times (D14 and D18) with either a negative control (100 μmol/L, miRNA inhibitor negative control Dharmacon) or anti-miR-138-5p (100 μmol/L, miR-138-5p inhibitor Dharmacon). **c**-**g** Haemodynamic measurements were performed by right heart catheterization (*n* = 6 rats per group). In vivo inhibition of miR-138-5p decreased right ventricle (RV) systolic pressure (RVSP) (**c**), decreased RV hypertrophy (Fulton index) (**d**) and increased cardiac output (CO) (**e**), consequently decreasing total pulmonary vascular resistance (evaluated by the RVSP/CO ratio) (**f**) and systemic carotid pressure (*n* = 3 rats per group) (**g**) *P < 0.05, **P < 0.01, ***P < 0.001

In 2011, Courboulin et al. found that miR-138-5p is overexpressed in the lungs and pulmonary arteries (PAs) isolated from iPAH patients compared to those isolated from non-PAH patients, and it is also overexpressed in the lungs of rats with MCT-PH [6]. Here, we confirmed that miR-138-5p expression was increased in the lungs of rats with MCT-PH (Fig. 1a, left panel), while miR-138-5p expression was unchanged in the right and left ventricles (RV and LV) of the same rats with MCT-PH (Fig. 1a middle and right panel). This finding suggests a crucial role of miR-138-5p in pulmonary vascular remodelling in MCT-exposed rats.

### In vivo inhibition of miR-138-5p reduces the development of PH in MCT-exposed rats

We assessed the biological impact of the inhibition of miR-138-5p on the development of established PH in rats exposed to MCT (60 mg/kg). Two weeks after MCT exposure, once PH was established, we nebulized anti-miR-138-5p at 100 μmol/L (2 times, D14 and D18) (Fig. 1b). Three weeks after MCT injection, closed chest right heart catheterization showed a significant increase in RVSP in MCT-anti-miR-Control vs saline treated rats (90 ± 7.1 vs 27 ± 2.7 mmHg) (Fig. 1c) associated with a strong increase in Fulton index (0.63 ± 0.004 vs 0.24 ± 0.009, reflecting RV hypertrophy) (Fig. 1c), a decrease in cardiac output (90 ± 3.9 vs 50 ± 2 ml/min) (Fig. 1d) and a strong increase in pulmonary vascular resistance evaluated by the ratio RVSP/CO (2 ± 0.2 vs 0.34 ± 0.02) (Fig. 1e). These results demonstrated, as expected, the development of severe PH in MCT + anti-miR-Control rats. Interestingly, the inhibition of miR-138-5p, by nebulization of an anti-miR-138-5p in MCT-PH rats, starting at week 2 after MCT exposure, strongly reduced PH severity as demonstrated by the decrease in RVSP (51 ± 7.3 vs 90 ± 7.1 mmHg), by the decrease in RV hypertrophy (0.47 ± 0.039 vs 0.63 ± 0.004), the improvement of cardiac output (80 ± 11.5 vs 50 ± 3.9 ml/min) and an important reduction in pulmonary vascular resistance (RVSP/CO, 0.7 ± 0.15 vs 2 ± 0.2) (Fig. 1b-e) compared to those in the MCT + anti-miR-Control-treated rats. To evaluate the effect of anti-miR-138-5p treatment on systemic circulation, we measured the systemic pressure in the carotid artery. As indicated in Fig. 1f, the systemic pressure was unchanged among the experimental conditions (Fig. 1f).

Three weeks after MCT exposure, the survival rate was the same between the MCT + anti-miR-Control and MCT + anti-miR-138-5p groups (not shown).

**Fig. 2:**
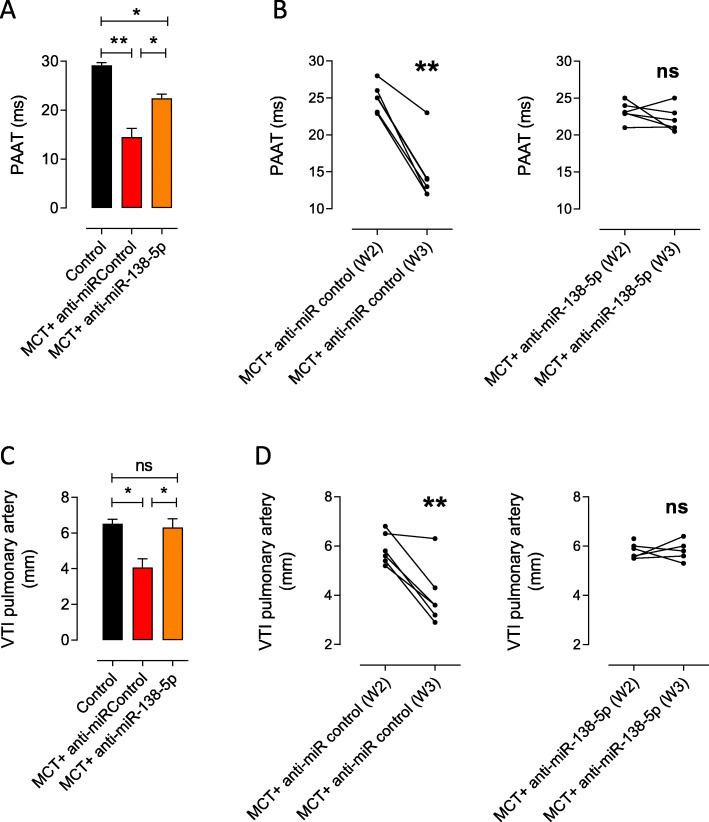
In vivo *inhibition of miR-138-5p stops the alteration of pulmonary arterial flow as shown by echocardiography.* In rats with MCT-PH, **a** quantification of the pulmonary acceleration time (PAAT) 3 weeks after MCT injection (*n* = 6 per group). **b** Evolution of the PAAT in MCT-exposed rats before (2 weeks) and after anti-miR (anti-miR-Control and anti-miR-138-5p) treatment (3 weeks). **c** Quantification of the pulmonary artery velocity time integral (VTI-PA) 3 weeks after MCT injection. **d** Evolution of the VTI-PA in MCT-exposed rats before (2 weeks) and after anti-miR treatment (3 weeks). *P < 0.05, **P < 0.01

In addition to the improvement of PH in the anti-miR-138-5p-treated, MCT-exposed rats, RV echocardiography measurements revealed significantly higher pulmonary artery acceleration time (PAAT) (Fig. 2a-b) and higher velocity time integral (VTI) of the pulmonary artery (VTI PA) (Fig. 2c-d) in the anti-miR-138-5p group compared with those in the anti-miR-Control group 3 weeks after MCT exposure. Interestingly, comparing the PAAT and VTI PA at 2 weeks and 3 weeks post MCT exposure, we found that the anti-miR-138-5p treatment stopped the worsening of PH (no further decrease in the PAAT and VTI PA values over time), while the anti-miR-Control treatment had no effect on PH progression (time-dependent decrease in the PAAT and VTI PA values) (Fig. 2b-d).

### Inhibition of miR-138-5p reduces pulmonary vascular remodelling and aberrant pulmonary cell proliferation in MCT-exposed rats

**Fig. 3:**
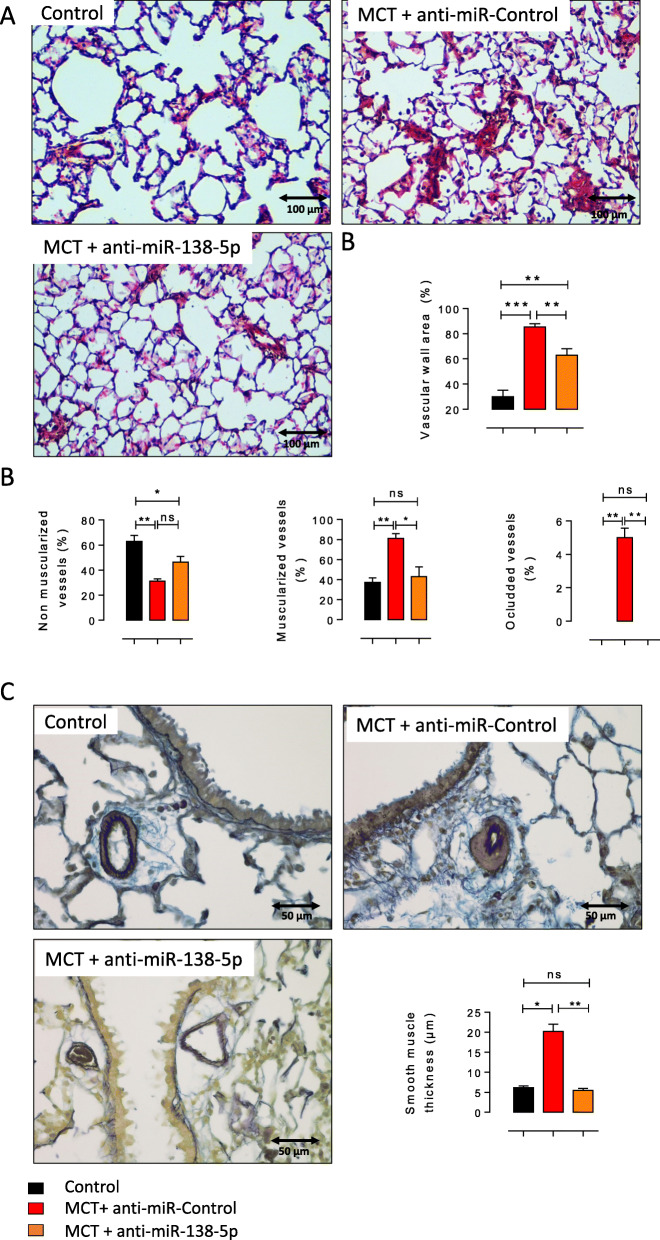
In vivo *inhibition of miR-138-5p reduces pulmonary vascular remodelling in rats with MCT-PH.***a** Representative haematein and eosin staining of the paraffin-embedded lung sections from the control, MCT + anti-miR-control, and MCT + anti-miR-138-5p groups. Scale bar = 100 μm (B) Vascular wall area (%) in the control, MCT + anti-miR-control, and MCT + anti-miR-138-5p groups (*n* = 5 different rats per condition). **b** Quantification of the percentage of nonmuscularized vessels (VWF-positive vessels, left panel), muscularized vessels (αSMA- and VWF-positive vessels, middle panel) and occluded vessels (right panel) (*n* = 6 different rats per condition). **c** Representative images of pulmonary arteries by Orcein staining (two elastic fibres) and quantification of the mean smooth muscle thickness (μm) of the pulmonary arteries from the Control, MCT + anti-miR-Control and MCT + anti-miR138–5p groups (*n* = 6 different rats per condition). *P < 0.05, **P < 0.01, ***P < 0.001

In addition to the decreased pulmonary vascular resistance, anti-miR-138-5p nebulization significantly reduced the vascular wall thickness compared with MCT + anti-miR-Control treatment (Fig. 3a-b). To further characterize pulmonary vascular remodelling, we analysed the muscularization of the small pulmonary vessels (< 30 μm) by immunostaining for α-SMA (α-smooth muscle actin, a smooth muscle marker) and VWF (Von Willebrand factor, an endothelium marker). MCT + anti-miR-Control-treated rats demonstrated a significantly decreased percentage of nonmuscularized vessels and a significantly increased number of muscularized vessels and occluded vessels compared to the control-treated rats (Fig. 3c). In the MCT + anti-miR-138-5p group, we found a significant reduction in the number of muscularized and occluded vessels (Fig. 3c). Finally, we measured the medial wall thickness (MWT) of the muscularized pulmonary arteries using Orcein staining to stain elastic fibres. We measured the MWT of the pulmonary vessels delimited by two elastic lamina (pulmonary artery). As illustrated in Fig. 3d, we found that the anti-miR-138-5p treatment normalized the MWT (Fig. 3d). Together, these results show that anti-miR138–5p treatment reduces vessel neomuscularization and media hypertrophy.

**Fig. 4:**
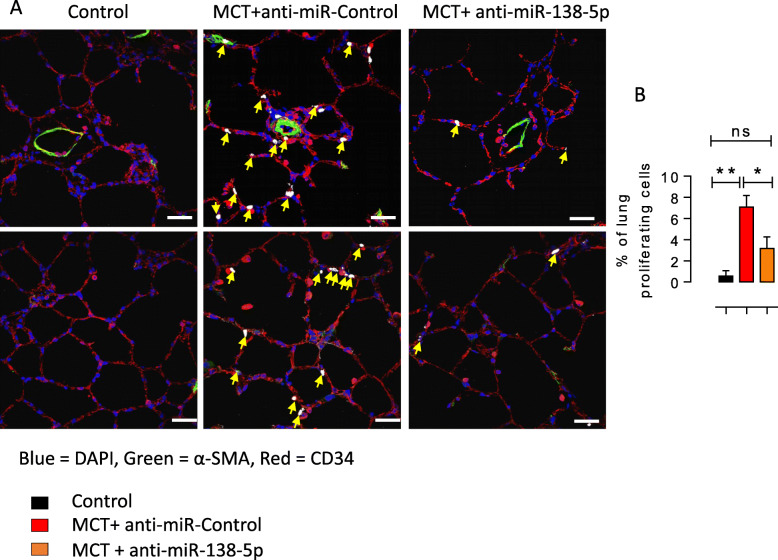
In vivo *inhibition of miR-138-5p reduces the pulmonary vascular cell proliferation in MCT-PH rats.***a** Immunofluorescence staining of frozen rat lung sections and confocal imaging with Click-iT 5-ethynyl-2′-deoxyuridine (EdU; white nuclei = EdU-positive nuclei = proliferating cells, yellow arrow) in combination with α-smooth muscle actin (α-SMA; in green, CD34; in red). Scale bar = 20 μm. The cells were counterstained with DAPI (blue). **b** Quantification of the percentage of proliferating cells in the lungs. (*n* = 6 different rats per condition). *P < 0.05, **P < 0.01

Moreover, anti-miR-138-5p nebulization reduced the percentage of proliferating cells in the lungs, as evaluated by EdU incorporation (Fig. 4d). As shown in Fig. 4d, most EdU-positive cells were also positive for CD34, demonstrating the nonmuscular nature of the proliferating EdU-positive cells in the lungs of the MCT + anti-miR-Control-treated rats (Fig. 4d).

### Inhibition of miR-138-5p reduces pulmonary inflammation and inflammatory cell infiltration in MCT-exposed rats

**Fig. 5:**
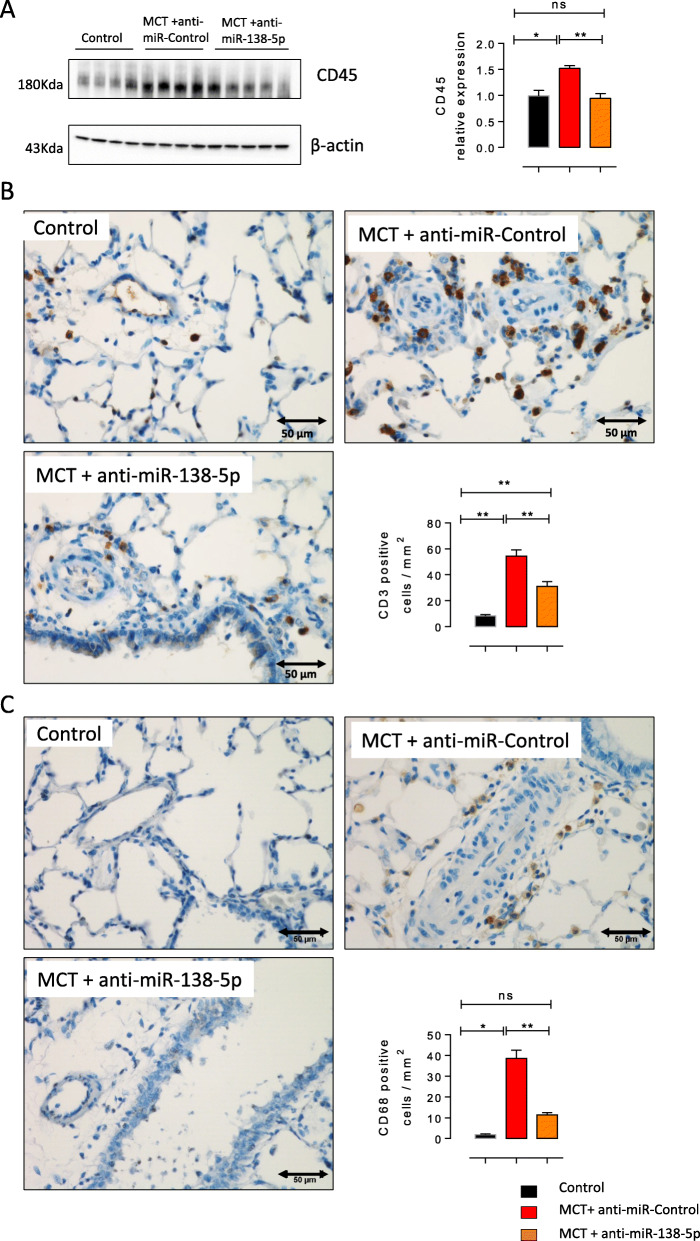
In vivo *inhibition of miR-138-5p reduces lung inflammation and inflammatory cell infiltration in MCT-PH rats.***a** CD45 (a pan-leucocyte marker) protein expression in the lungs was analysed by western blot analysis (*n* = 6 per group). Quantification of the CD45 protein expression in the lungs. (*n* = 6 different rats per condition). **b** Representative images of T lymphocyte (CD3-positive cells) accumulation and quantification of the mean number of T lymphocytes (CD3+ cells) per mm^2^ in the lung sections from the Control, MCT + anti-miR-Control and MCT + anti-miR138–5p groups (*n* = 6 different rats per condition). *P < 0.05, **P < 0.01, ***P < 0.001. **c** Representative images of macrophage (CD68-positive cells) accumulation and quantification of the mean number of macrophages (CD68+ cells) per mm^2^ in the lung sections from the Control, MCT + anti-miR-Control and MCT + anti-miR138–5p groups (*n* = 6 different rats per condition). *P < 0.05, **P < 0.01, ***P < 0.001

Western blot analysis of CD45 (a pan leukocyte marker) indicated a significant decrease in the lung inflammation in the MCT-anti-miR-138-5p-treated animals (Fig. 5a), as shown by HES staining. Immunostaining for CD3 (a T lymphocyte marker) showed that T lymphocyte infiltration into the lungs of rats with MCT-PH was significantly reduced in the MCT + anti-miR-138-5p-treated rats compared to the MCT + anti-miR-Control-treated rats (Fig. 5b). Finally, as indicated by the strong reduction in the number of CD68-positive (macrophage marker) cells in the lung sections from the MCT + anti-miR-138-5p-treated rats, anti-miR138–5p treatment greatly decreased the accumulation of macrophages in the lungs of the rats with MCT-PH (Fig. 5c).

### Inhibition of miR-138-5p restores lung KCNK3 and SLC45A3 expression

**Fig. 6:**
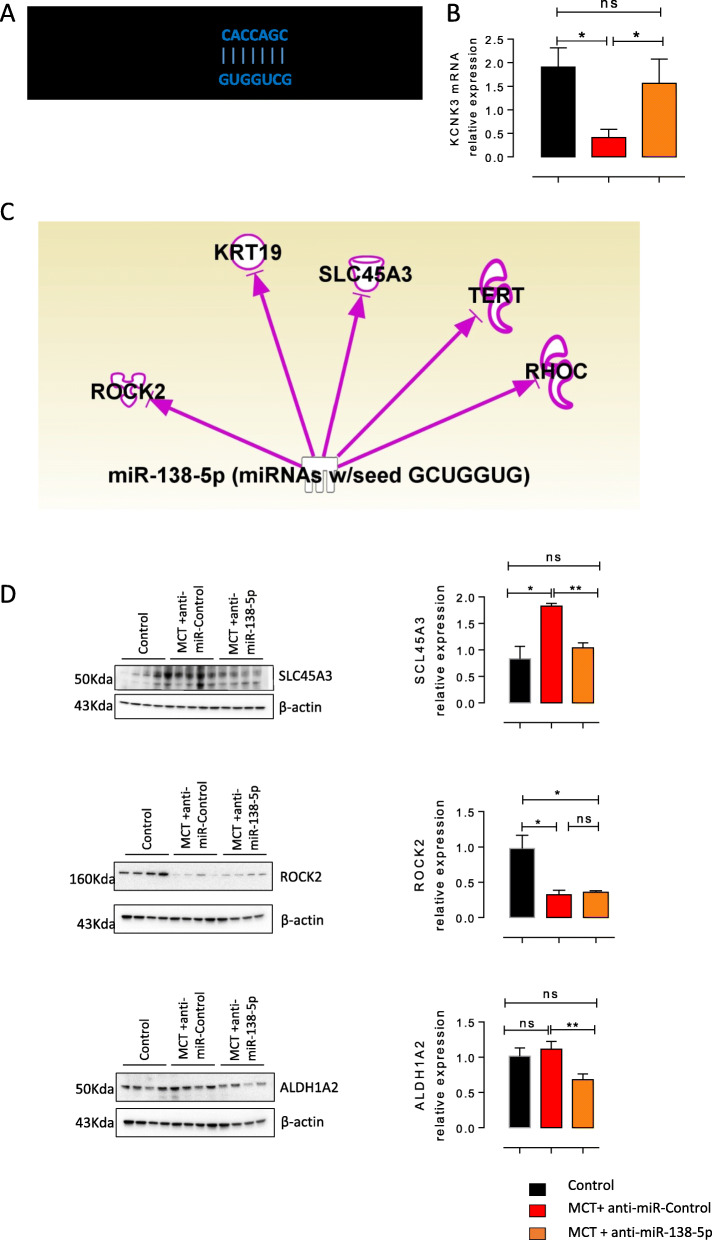
*Nebulized miR-138-5p restores the expression of KCNK3 and SLC45A3 in the lungs.***a** Diagram of the miR-138-5p putative binding site in the human KCNK3 3′ UTR *(B)* mRNA expression of KCNK3 in the lungs from the control rats, MCT- and anti-miRNA-control-treated rats and MCT- and anti-miR-138-5p-treated rats (*n* = 5 different rats per condition). **b** Network analysis using Ingenuity Pathway Analysis (IPA) software, with the following settings: confidence levels: experimentally observed; species: human, rat. This figure shows that miR-138-5p was shown to regulate the expression of several genes, including *SLC45A3 (solute carrier family 45 member 3), ALDH1A2 (aldehyde dehydrogenase 1 family member A2), ROCK2 Rho-associated coiled-coil containing protein kinase 2), TERT (telomerase reverse transcriptase), RHOC (Ras homologue family member C), VCAN (versican)* (**c**) SLC45A3, ALDH1A2 and ROCK2 lung protein expression analysed by western blot (*n* = 5 per group). Quantification of lung SLC45A3, ALDH1A2 and ROCK2 protein expression. (*n* = 6 different rats per condition). *P < 0.05, **P < 0.01, ***P < 0.001

We previously demonstrated the loss of KCNK3 expression in the lungs from rats with MCT-PH [13] and confirmed in the present study, and miR-138-5-p target sites within the 3′ UTR of KCNK3 mRNA, as predicted by nucleotide BLAST (Target Scan) (Fig. 6a). We next evaluated the consequences of in vivo miR-138-5p inhibition on KCNK3 expression at the mRNA level. Quantitative RT-PCR on the lungs from these animals revealed that the mRNA level of KCNK3 was strongly reduced in the MCT + anti-miR-Control group and restored to a level similar to that observed in the control animals in the MCT + anti-miR-138-5p group (Fig. 6b).

Since Target Scan indicates the theoretical regulation, we used Ingenuity Pathway Analysis (IPA) software to identify other genes that have been experimentally proven to be regulated by miR-138-5p. IPA is a Web-based mammalian biology knowledge base and a tool used for genomic data analysis, and we used the following settings: confidence levels: experimentally observed; species: human, rat.

With IPA, we found that miR-138-5p regulated several genes, including *SLC45A3* (solute carrier family 45 member 3), *ALDH1A2* (aldehyde dehydrogenase 1 family member A2), *ROCK2* (Rho-associated coiled-coil containing protein kinase 2), *TERT* (telomerase reverse transcriptase), *RHOC* (Ras homologue family member C), and *VCAN* (versican) (Fig. 6c). By western blot, we analysed the pulmonary protein expression of SLC45A3, ALDH1A2 and ROCK2. As shown in Fig. 5b, SLC45A3 was overexpressed in the MCT-anti-miR-Control-treated rats and normalized after treatment with anti-miR-138-5P (Fig. 6c-d). ALDH1A2 expression was similar in the MCT-anti-miR-Control group compared to the control group (Fig. 6c-d). However, anti-miR-138-5p treatment reduced the expression of ALDH1A2 in the lungs (Fig. 6c-d). Finally, ROCK2 protein expression was reduced in the MCT-anti-miR-Control- and MCT-anti-miR-138-5p-treated animals compared to the control animals. The results suggest that miR-138-5p in the MCT rats could also act as a regulator of KCNK3 and SLC45A3 and identify SLC45A3 as a possible new actor involved in the pathobiology of MCT-PH.

## Discussion

In the present study, we confirmed that the in vivo inhibition of miR-138-5p reduces the development of established MCT-PH and reduces pulmonary vascular remodelling, pulmonary inflammation and aberrant pulmonary vascular cell proliferation. In vivo miR-138-5p inhibition normalizes lung KCNK3 and SLC45A3 expression.

Bioinformatics-based (Target Scan®) analyses identified *KCNK3* as a direct target of miR-138-5p. Consistent with these bioinformatics tools, Liu and colleagues, using a luciferase assay, demonstrated that miR-138-5p may directly target *KCNK3* in human PASMCs. These authors also found that the inhibition of miR-138-5p in human PASMCs significantly increased the expression of *KCNK3* mRNA [8]. Here, we found that the in vivo inhibition of miR-138-5p by intratracheal nebulization restores the loss of KCNK3 mRNA expression in the lungs.

MiR-138-5p was shown to promote the proliferation and migration of vascular smooth muscle cells in diabetic mice by suppressing the expression of SIRT1 [23], the inactivation of which favours PH [24]. MiR-138-5p induces the phosphorylation of ERK1/2, leading to the cleavage of procaspase-3 and the upregulation of Bcl-2 [8] and promoting the apoptotic status of human PASMCs. Moreover, in cultured human PASMCs, miR-138-5p overexpression was demonstrated to reduce KCNK3 expression [8].

MiR-138-5p contributes to cell proliferation and invasion of bladder cancer cells by targeting [25]. In pancreatic cancer cells, miR-138-5p is suggested to play an important role in the regulation of cell growth by acting on FOXC1 [26]. We found that the in vivo inhibition of miR-138-5p reduced the aberrant proliferation of pulmonary cells in rats with MCT-PH, and these cells were mainly positive for CD34, an endothelial cell marker, suggesting that anti-miR-138-5p treatment reduces the aberrant proliferation of microvascular endothelial cells. Indeed, under a hypoxic conditions, miR-138-5p is increased in endothelial cells in association with the development of endothelial dysfunction.

However, CD34 is also used as a marker of haematopoietic stem or progenitor cells and nonhaematopoietic cells, including vascular endothelial progenitors 1 [27]. In rats with MCT-PH, a population of PW1+/CD34+ pulmonary progenitor cells are recruited to the lung compared to the control rats, and this population contributes to pulmonary vessel neomuscularization [28]. According to these observations, we hypothesized that anti-miR138–5p also reduced pulmonary vessel muscularization by decreasing progenitor cell recruitment.

In the present study, we observed a reduction in lung inflammation and a reduction in macrophage and T lymphocyte lung infiltration in the MCT-anti-miR138–5p-treated rats. In neurons, miR-138-5p is involved in the progression of Parkinson’s disease by regulating the inflammatory response via the SIRT1 axis [29]. Moreover, the downregulation of miR-138-5p protects chondrocytes from IL-1β-induced inflammation [30]. MiR-138-5p was found to reduce the expression of the PD-1 receptor (programmed death-ligand 1 receptor) [31], which is an important modulator of T cell function [32]. MiR-138–5p is also known to be involved in the downregulation of CTLA-4 (cytotoxic T-lymphocyte-associated protein 4) and to indirectly regulate macrophage function via engagement on antigen presenting cells [33]. These inhibitory effects of anti-miR-138-5p treatment on the immune checkpoint also contribute to alleviating PH in the MCT-induced model.

Courboulin et al. previously described that miR-138-5p is overexpressed in the lungs of iPAH patients [6]. MiR-138-5p expression is increased in human PASMCs exposed to hypoxia [7]. Finally, the in vivo inhibition of miR-138-5p was shown to reduce the development of severe experimental PAH by regulating the expression of MCU1 (mitochondrial Ca^2+^ uniportor 1) and CREB (cAMP response element binding) [17]. In the present study, we confirmed that the in vivo inhibition of miR-138-5p limits the exacerbation of MCT-PH at the pulmonary vascular and cardiac levels.

MiR-138-5p and other miRNAs regulate several genes (including *KCNK3* and *SLC45A3*) which could potentially have consequences for the development of PH. Recently, Babicheva A and colleagues recently demonstrated that miR-138-5p regulated the global K^+^ current in control human PASMCs and that a miR-138-5p antagomir partly restored the K^+^ function in iPAH PASMCs [34]. Moreover, miR-138-5p is described to cause endothelial dysfunction and impair tube formation [35].

SLC45A3 (solute carrier family 45 member 3) is a proton-associated sugar transport that regulates the transport of glucose or other sugar or metal ions [36]. SLC45A3 overexpression was reported in aggressive prostate cancer progression [37]. Here, we found, for the first time, that SLC45A3 was overexpressed in the lungs of rats with MCT-PH, suggesting a putative role for SLC45A3 in PH development. However, SLC45A3 expression and function in the lung are not completely understood. Hence, further studies are required to delineate the role of SLC45A3 in pulmonary vascular cells and its role in PAH.

## Conclusions

In conclusion, our results confirm that in vivo inhibition of miR-138-5p could be a therapeutic option to reduce PH severity and that restoration of pulmonary KCNK3 and SLC45A3 expression could contribute to disease alleviation.

## Data Availability

All data generated or analyzed during this study are included in this article.

## Abbreviations

PAH: Pulmonary arterial hypertension
PH: Pulmonary hypertension
MCT: Monocrotaline
RV: Right ventricular
PASMC: Pulmonary arterial smooth muscle cells
MWT: Medial wall thickness
RVSP: Right ventricular systolic pressure
CO: Cardiac output
PVR: Pulmonary vascular resistance
PAAT: Pulmonary artery acceleration time
K2P: Two-pore K^+^ channel
